# The effects of alpha 1-adrenoceptor blockade and angiotensin converting enzyme inhibition on central and brachial blood pressure and vascular reactivity: the doxazosin–ramipril study

**DOI:** 10.1007/s00380-016-0924-9

**Published:** 2016-11-24

**Authors:** Andreas Jekell, Majid Kalani, Thomas Kahan

**Affiliations:** 1 Division of Cardiovascular Medicine, Department of Clinical Sciences, Danderyd Hospital, Karolinska Institutet, Stockholm, Sweden; 2 Department of Cardiology, Danderyd University Hospital Corp, Stockholm, Sweden

**Keywords:** Arterial stiffness, Blood pressure, Endothelial function, Renin-angiotensin-aldosterone system, Treatment

## Abstract

We aimed to study whether inhibition of the renin–angiotensin–aldosterone system has effects on vascular structure and function beyond the effects on blood pressure reduction alone. Patients with mild-to-moderate hypertension (*n* = 61, age 54 ± 12 years, 34% women) received the angiotensin converting enzyme inhibitor ramipril 10 mg or the alpha 1-adrenoceptor blocker doxazosin 8 mg double-blind for 12 weeks. Aortic blood pressure, pulse wave velocity, and augmentation index were assessed by applanation tonometry. Endothelial function was studied by forearm post-ischemic flow mediated vasodilatation and by pulse wave analysis with beta 2-adrenoceptor agonist stimulation. Skin microvascular reactivity was assessed by laser Doppler fluxmetry and iontophoresis. Treatment with doxazosin or ramipril reduced aortic and brachial blood pressures (all *P* < 0.001), with greater reductions in aortic than brachial systolic blood pressures (*P* = 0.021) and aortic/brachial pulse pressure ratio (*P* = 0.005). Compared to doxazosin, ramipril reduced carotid-femoral and carotid-radial pulse wave velocity (both *P* < 0.05). Forearm endothelial dependent and independent vasodilatation, assessed by post-ischemic flow mediated vasodilatation and glyceryl trinitrate, and by pulse wave analysis remained unchanged by both doxazosin and ramipril. In addition, skin microvascular endothelial dependent (acetylcholine) and independent vasodilatation (sodium nitroprusside) remained unchanged. In conclusion, ramipril reduced indices of aortic stiffness, suggesting that angiotensin converting enzyme inhibitor therapy may have effects beyond blood pressure reduction. However, treatment did not appear to influence endothelial function. Evidence of endothelial dysfunction and its possible improvement by antihypertensive treatment might require more advanced hypertension.

This study is registered at ClinicalTrials.gov (NCT02901977) and at EudraCT (# 2007-000631-25).

## Introduction

Hypertension is characterized by an increased sympathetic vascular tone, early vascular remodelling of small resistance arteries, and impaired endothelial function [1–3]. Thus, hypertension increases pulsatile load on the vasculature, which induces aortic stiffness and increases pulse wave velocity (PWV). This further changes pulse wave reflection, resulting in augmentation of the reflective retrograde pulse wave and an increase in central aortic blood pressure (BP). Compared to the peripheral BP, values on central BP, augmentation index (AIx), aortic stiffness, and arterial stiffness of different vascular regions give additional information about future cardiovascular events [4–6]. Furthermore, aortic dilatation, increased aortic stiffness, AIx, and central pulse pressure are associated with endothelial dysfunction of conduit arteries [7–10], and endothelial dysfunction is associated with an increased risk of cardiac events [11].

Antihypertensive treatment plays a key role to reverse hypertension induced structural vascular changes. Activation of the renin–angiotensin–aldosterone system (RAAS) increases the formation of angiotensin II, which mediates vasoconstriction and promotes inflammation, endothelial dysfunction, oxidative stress, and structural vascular changes [12]. Angiotensin converting enzyme (ACE) inhibitors and angiotensin receptor blockers may have effects beyond BP lowering to reverse vascular remodelling [13–16]. However, the reported effects of blocking the RAAS on endothelial function are conflicting and the mechanisms remain to be clarified [17–19]; and systematic comparisons to blocking sympathetic vasoconstrictor nerve activity by alpha 1-adrenoceptor blockers are not present [20, 21]. Furthermore, ACE inhibitors appear to reduce arterial stiffness independent of their ability to reduce BP, but it remains unclear whether they are superior to other antihypertensive drug classes; and the effects of alpha 1-adrenoceptor blockers have not been well studied [22, 23]. ACE inhibitors have been proposed to improve cardiovascular outcome in high-risk patients beyond the effects of BP reduction [24, 25]. However, the possible additional effects of various antihypertensive drug classes on clinical outcome beyond their reduction in BP remain to be clarified [26].

This study aimed to investigate the possible influence of the RAAS on indices of central and peripheral vascular structure and function, and on endothelial function beyond the effects on blood pressure. Thus, we compared the effects of blocking the RAAS by the ACE inhibitor ramipril to reducing noradrenergic sympathetic vascular tone by the alpha 1-adrenoceptor blocker doxazosin in patients with uncomplicated mild-to-moderate hypertension. We assessed central BP and indices of aortic stiffness by pulse wave analysis, and endothelial function was examined by several methods in the forearm skeletal muscle and in the skin microcirculation, to reflect both conduit and resistance artery function.

## Materials and methods

### Study design and subjects

Women and men 18 years of age or older with mild-to-moderate primary hypertension were considered eligible for the Doxazosin–Ramipril Study if their office BP, obtained as a mean of 2 or more measurements by standard techniques in the supine position with an appropriate cuff size and mercury sphygmomanometer, was >140 mm Hg systolic and/or >90 mm Hg diastolic. Ambulatory BP monitoring had been performed in most patients to confirm the diagnosis of hypertension. All subjects were previously untreated or free from antihypertensive or other drug therapy with potential influence on BP, vascular, or endothelial function for at least 4 weeks. Secondary hypertension was ruled out by physical examination and routine biochemical examinations. Patients with BP >180/110 mm Hg, coronary artery disease, congestive heart failure, or atrial fibrillation or flutter, diabetes mellitus, or chronic kidney disease were excluded.

Randomization to double-blind treatment with doxazosin 4 mg od or ramipril 5 mg od for 2 weeks with forced titration to doxazosin 8 mg od and ramipril 10 mg od for an additional 10 weeks was performed by a computer-generated list in blocks of four, stratified by sex. The two study drugs had identical appearance and were provided in identical containers for each patient, with the contents blinded for both patient and investigator (Apoteket Produktion & Laboratorier AB, Stockholm, Sweden).

The study included 71 patients (63 with never treated hypertension); 10 patients (5 women and 5 men) discontinued due to reported side-effects (8 on doxazosin and 2 on ramipril). No serious side-effects were reported. Thus, we here report on 61 patients (56 were never treated for hypertension, and 3 previously treated with amlodipine, 1 with metoprolol, and 1 with enalapril). All participants achieved the targeted 10 weeks of treatment with 8 mg doxazosin od or 10 mg ramipril od. Only 2 women on ramipril and 1 on doxazosin were premenopausal (i.e. <45 years of age), and no participant used systemic used hormone replacement therapy.

Patients arrived in the morning after fasting overnight for the investigations at weeks 0 and 12. They were undertaken on 2 consecutive days on both occasions, to avoid pharmacological interference of the examination protocols on vascular function. The patients were asked to take their study medication approximately 2 h before the investigations to achieve peak plasma concentrations, but to refrain from caffeine containing beverages, fruit juices or vitamin C, and any other medication (including thrombocyte inhibitory drugs for 7 days and non-steroid anti-inflammatory drugs for 48 h). Examinations were performed in the supine position following a 20 min period of rest in a quiet room kept at 21–24 °C constant temperature.

### Blood pressure measurements and pulse wave analysis

For the purpose of the vascular function studies, brachial BP was obtained in the supine position by an oscillometric device (OMRON 705IT, OMRON Healthcare Co., Ltd. Kyoto Japan) on the right arm with an appropriately sized cuff as a mean of three readings 1 min apart.

Applanation tonometry was performed using a SphygmoCor device (AtCor Pty Ltd, West Ryde, NSW, Australia) according to current recommendations [27]. Radial artery waveforms were calibrated using brachial systolic and diastolic BP measured in the same arm (vide supra), the central aortic waveform was calculated by device software using the generalized transfer function, and central BP values were derived. AIx was measured through the software. Recordings were then repeated at the level of the common carotid artery and the femoral artery, and PWV was calculated from the direct (carotid-to-radial and carotid-to-femoral) path length.

Pulse pressure was calculated as systolic minus diastolic BP. Mean arterial pressure was calculated as diastolic BP + 1/3 *x* pulse pressure. Body mass index (in kg/m^2^) was calculated as weight/height^2^.

### Assessment of endothelial function

Endothelium dependent flow mediated vasodilatation (FMD) was measured by ischemia induced reactive hyperaemia in the non-dominant arm according to current recommendations [28]. Vasodilatation was induced by inflation of a pneumatic tourniquet placed around the forearm to a pressure of 250 mm Hg for 5 min, followed by release. Brachial artery diameter was measured proximal to the tourniquet by a Vivid 7 Dimension (GE Medical System, Horten, Norway) ultrasound device and a 9 MHz linear transducer. All images were stored for later analyses. The mean values of 3 measurements of arterial diameter performed at end diastole were calculated at rest and at 30, 60, and 90 s after cuff release. The maximal relative increase in diameter was taken as a measure of FMD. After a period of at least 10 min to regain stable resting conditions, 0.4 mg glyceryl trinitrate (Nitrolingual, G Pohl-Boskamp GmbH & Co KG, Hohenlockstedt, Germany) given as sublingual spray was used to assess endothelium-independent vasodilatation. Relative changes in artery diameter were calculated from rest to 4 min following drug administration. To better assess endothelial function, we also calculated the endothelial function index by the ratio of the maximum relative increase in flow by reactive hyperaemia to glyceryl trinitrate, as previously proposed [29]. We calculated local shear stress, an important stimulus for FMD, as 8 *x* µ *x* blood flow velocity/baseline brachial artery diameter, where µ is blood viscosity, which was assumed to be 0.035 dyne *x* s/cm^2^ [30]. The inter-assay coefficient of variation for FMD in our laboratory is 15% (*n* = 20).

Endothelial function was also assessed by beta 2-adrenoceptor agonist induced changes in the pulse waveform [31]. Radial artery pulse waves were recorded by applanation tonometry, and the maximal systolic peak and the reflected waves were identified by the calculations of the first and second derivatives of the pulse curve. The relative height of the diastolic reflected wave (i.e., the reflection index) was used as an index of endothelial function [32] After a recording under resting conditions, 0.25 mg terbutaline (Bricanyl, AstraZeneca, Mölndal, Sweden) was given subcutaneously in the upper forearm and the pulse waveform was again evaluated after 15 and 20 min. The maximal relative change was used. A large reduction of the reflection index indicates a good response.

Endothelium dependent and independent forearm skin microvascular vasodilatation was assessed by laser Doppler fluxmetry and transcutaneous iontophoretic administration (Periflux system 5000, PF 5010 LDPM Unit, PF5010 Temp Unit, and 481-1 Single Probe, Perimed, Järfälla, Sweden) of small amounts of acetylcholine (Sigma-Aldrich AB, Stockholm, Sweden) and sodium nitroprusside (Hospira, Inc., Lake Forest, IL, USA) for 60 s, as described previously [33]. Skin microvascular peak flux was recorded continuously up to 16 min after iontophoresis, and is expressed in arbitrary units. We also determined maximum skin microvascular hyperaemia by peak flux induced by local heating of forearm skin to 44 °C for 6 min.

### Biochemistry

Routine biochemistry was analysed by standard procedures from fasting blood samples obtained into Vacutainer tubes (Becton–Dickinson Co. Cedex, Meylan, France) on ice from an indwelling antecubital venous catheter after 20 min of supine rest.

### Statistics

The co-primary outcomes in the Doxazosin–Ramipril Study were changes in endothelial function assessed by FMD and in haemostatic function measured by the generation of thrombin–antithrombin complex (those results will be presented elsewhere). A meta-analysis indicates a 1% increase in FMD to be associated with a 13% lower risk for a future cardiovascular event, after adjustment for confounding risk factors [34]. Thus, assuming 2 alpha 0.05 and beta 0.80, we calculated a study population of 2 × 24 subjects sufficient to detect a 0.6% difference in FMD by treatment between the two study groups (with an SD of 0.72% in our laboratory) sufficient to ascertain a clinically important difference; and 2 × 26 subjects sufficient to detect a clinically relevant 0.4 mg/l difference in thrombin–antithrombin complex by treatment between the two groups (with an SD of 0.5 mg/l in our laboratory). Assuming that patients may be lost due to withdrawals, side-effects, technical, or analytical problems, we intended to include 70 subjects to have at least 60 completed patients and to ensure a sufficient number of evaluable patients.

Data are presented as mean values ± SD (or mean values ± SEM for calculated differences) or medians and interquartile range, as appropriate. Skewed variables were logarithmically transformed. Group comparisons were made by the analysis of variance or by multivariate analysis of variance. Multiple linear regression analysis was used to assess effects of treatment. To account for potential confounders concerning PWV and AIx, the initial multivariate mode always included baseline mean arterial pressure, heart rate, height, and age; gender did not affect the results. All results were similar if mean arterial pressures at week 12 instead of baseline values were included in the model. All statistical tests were 2-sided and carried out to a significance level (*P*) of 0.05. The statistical program used was JMP version 11.1 (SAS Institute Inc., Cary, NC, USA).

## Results

### General

Background characteristics of the two study groups are presented in Table 1. The two study groups were comparable and there were no significant differences between the groups. Outpatient office BP values at inclusion were 150 ± 8/92 ± 10 and 155 ± 9/94 ± 7 mm Hg in patients eventually randomized to doxazosin and ramipril, respectively. Aortic and brachial BP and heart rate values (recorded in the laboratory at the time of the investigation), and indices of central vascular function were also similar in the two groups (Table 2). Aortic and brachial pulse pressures were 49.5 ± 9.6 and 59.0 ± 8.1 mm Hg (*P* < 0.001) in the doxazosin group, and 51.4 ± 13.6 and 60.3 ± 9.7 mm Hg (*P* < 0.001) in the ramipril group, respectively.

**Table 1:** Baseline characteristics

	Doxazosin	Ramipril	All
*n*	28	33	61
Male/female (n)	20/8	20/13	40/21
Age, years (range)	53.5 ± 11.3 (26-75)	53.7 ± 13.3 (23-70)	53.6 ± 12.3 (23-75)
Smoker (*n*)	2	2	4
Height (cm)	176.4 ± 7.6	173.5 ± 9.3	174.9 ± 8.6
Body mass index (kg/m^2^)	27.8 ± 5.4	25.8 ± 3.8	26.7 ± 4.7
Office systolic blood pressure (mm Hg)	150.5 ± 7.9	154.8 ± 9.3	152.5 ± 8.9
Office diastolic blood pressure (mm Hg)	92.2 ± 9.8	93.57 ± 7.1	92.9 ± 8.4
Baseline systolic blood pressure (mm Hg)	148.0 ± 10.9	148.3 ± 11.3	148.2 ± 11.1
Baseline diastolic blood pressure (mm Hg)	89.0 ± 10.3	88.0 ± 8.1	88.5 ± 9.1
Heart rate (min^−1^)	59 ± 9	62 ± 8	61 ± 8
Total cholesterol (mmol/L)	5.4 ± 1.2	5.4 ± 0.9	5.4 ± 1.1
Fasting plasma glucose (mmol/L)	5.5 ± 0.6	5.3 ± 0.5	5.4 ± 0.6
Creatinine (µmol/L)	78.6 ± 13.5	74.5 ± 14.6	76.5 ± 14.1
Haematocrit (%)	41.1 ± 3.2	41.3 ± 2.4	41.2 ± 2.8

Data presented as mean values ± SD, if not otherwise stated

**Table 2:** Treatment effects on blood pressure and vascular function

	Week	Doxazosin	Ramipril	*P* by repeated-measures ANOVA	
SBP br (mm Hg)	0	148.0 ± 11.0	148.3 ± 16.3	Time	<0.001
	12	142.3 ± 12.1	136.2 ± 11.6	Group	0.27
				Time × group	0.030
SBP ao (mm Hg)	0	140.3 ± 12.9	139.2 ± 15.8	Time	<0.001
	12	131.7 ± 14.8	124.7 ± 13.3	Group	0.19
				Time × group	0.039
DBP br (mm Hg)	0	89.0 ± 10.3	88.0 ± 8.1	Time	<0.001
	12	84.6 ± 10.3	80.1 ± 8.7	Group	0.21
				Time × group	0.073
DBP ao (mm Hg)	0	90.9 ± 10.0	87.8 ± 7.5	Time	<0.001
	12	85.2 ± 10.5	80.8 ± 7.1	Group	0.058
				Time × group	0.35
Heart rate (min^-1^)	0	58.9 ± 7.6	61.9 ± 8.1	Time	0.79
	12	58.8 ± 9.7	61.3 ± 7.4	Group	0.14
				Time × group	0.90
PWV car-fem (m/s)	0	8.5 ± 1.5	8.9 ± 2.0	Time	0.070
	12	8.3 ± 1.7	8.4 ± 1.9	Group	0.42
				Time × group	0.037
PWV car-rad (m/s)	0	8.7 ± 1.5	9.1 ± 1.0	Time	0.38
	12	8.7 ± 1.2	8.4 ± 1.1	Group	0.43
				Time × group	0.034
Augmentation index (%)	0	29.3 ± 10.4	30.7 ± 13.6	Time	0.37
	12	27.1 ± 11.4	26.8 ± 12.1	Group	0.78
				Time × group	0.37
PP ao/PP br	0	0.83 ± 0.12	0.85 ± 0.14	Time	0.005
	12	0.81 ± 0.14	0.78 ± 0.12	Group	0.72
				Time × group	0.21
PWV car-fem/PWV car-rad	0	0.98 ± 0.18	0.97 ± 0.19	Time	0.55
	12	0.94 ± 0.18	1.02 ± 0.22	Group	0.38
				Time × group	0.39

**Fig. 1:**
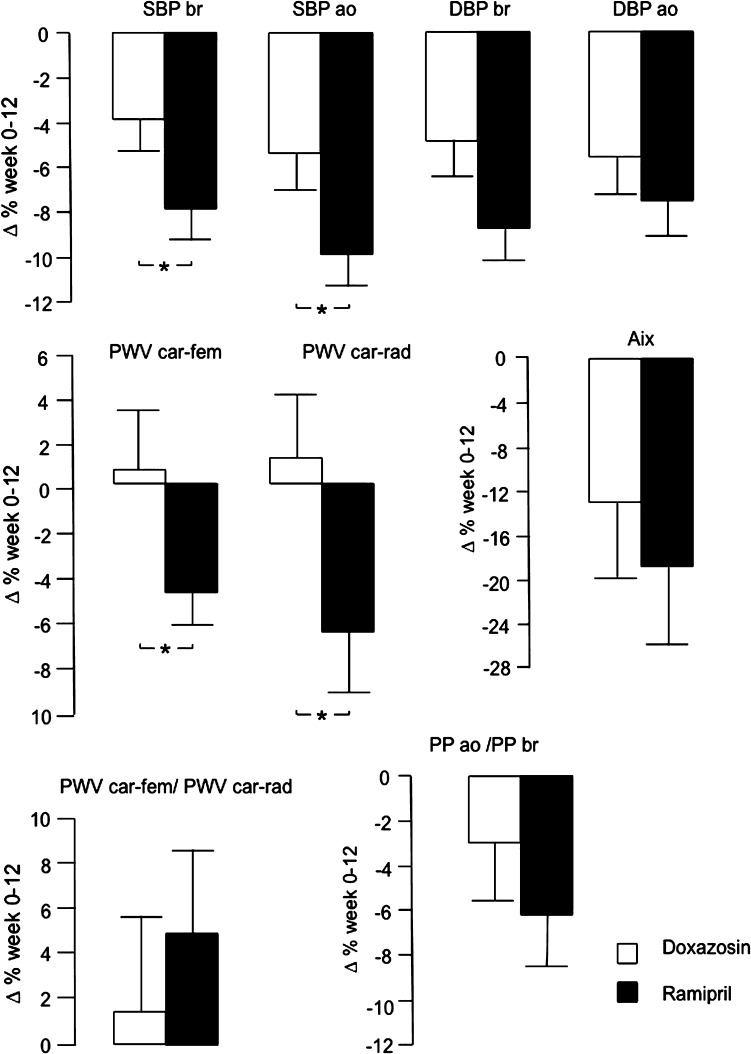
Relative changes (mean values ± SEM) in BP and vascular function by treatment. Significant treatment induced changes between groups are shown as *P < 0.05. Further statistical evaluation is presented in Table 2

Mean values ± SD at week 0 and 12 for 27–33 subjects in each treatment group, including all subjects with valid measurements at week 0 or 12. Relative differences (∆%) for paired observations are presented in Fig. 1. *P* denotes significant changes by repeated-measures MANOVA, where PWV car-fem, PWV car-rad, and augmentation index were adjusted for mean arterial pressure, heart rate, height, and age; gender did not affect the results

*SBP br* brachial systolic blood pressure, *SBP ao* aortic systolic blood pressure, *DBP br* brachial diastolic blood pressure, *DBP ao* aortic diastolic blood pressure, *PWV car-fem* carotid-femoral pulse wave velocity, *PWV car-rad* carotid-radial pulse wave velocity, *PP ao/PP br* aortic pulse pressure/brachial pulse pressure

### Effects on central and peripheral BP by treatment

Antihypertensive drug treatment reduced aortic and brachial BP in both study groups (Table 2; Fig. 1). Of note, the changes in aortic and brachial systolic BP were greater by ramipril than by doxazosin (Table 2; Fig. 1). Drug treatment reduced aortic systolic BP more than brachial BP (*P* = 0.021) with no difference between doxazosin and ramipril.

Drug treatment reduced aortic pulse pressure more than brachial pulse pressure (Fig. 1). The changes in aortic and brachial pulse pressure were −2.6 ± 5.7 (*P* = 0.030) and −1.4 ± 6.0 (*P* = 0.22) mm Hg for doxazosin, and −7.5 ± 8.7 (*P* < 0.001) and −4.2 ± 8.2 (*P* = 0.006) mm Hg for ramipril, respectively. Although the absolute changes in aortic (but not brachial) pulse pressure were greater (*P* = 0.016) in the ramipril group, the changes in the aortic to brachial pulse pressure ratios were similar in the two groups (Table 2).

### Effects on vascular function by treatment

Compared to doxazosin, ramipril reduced carotid-femoral PWV and carotid-radial PWV (also when mean arterial pressure, heart rate, height, age, and gender were considered) (Table 2; Fig. 1). There was no difference between the doxazosin and ramipril groups in the ratio carotid-femoral to carotid-radial PWV (Table 2; Fig. 1). AIx was numerically reduced in both study groups, although this did not reach significance (Table 2; Fig. 1).

### Effect on endothelial function by treatment

Data on brachial artery diameter and function are presented in Table 3. Indices of gross endothelial dependent and independent vasodilatation, assessed by forearm vascular function in response to post-ischemic FMD and glyceryl trinitrate, and by the reflection index derived from pulse wave analysis and beta 2-adrenoceptor agonist stimulation, remained unchanged by treatment with doxazosin or ramipril (Table 4). In addition, endothelial dependent and independent vasodilatation assessed in the skin microcirculation remained unchanged by treatment (Table 5).

**Table 3:** Forearm circulatory variables before and during post-ischemic hyperaemia

	Doxazosin	Ramipril	*P* by repeated-measures ANOVA	
Baseline brachial artery diameter (mm)				
Week 0	3.87 ± 0.51	3.70 ± 0.69	Time	0.36
Week 12	3.88 ± 0.52	3.78 ± 0.64	Group	0.34
			Time × group	0.52
Baseline mean flow velocity (cm/s)				
Week 0	5.2 ± 3.1	4.4 ± 3.1	Time	0.31
Week 12	5.5 ± 2.7	4.9 ± 2.8	Group	0.29
			Time × group	0.73
Hyperaemic mean flow velocity (cm/s)				
Week 0	37.2 ± 9.8	38.2 ± 8.7	Time	0.96
Week 12	41.3 ± 20.7	32.8 ± 7.5	Group	0.14
			Time × group	0.07
Baseline mean flow (ml/min)				
Week 0	61.8 ± 39.2	52.2 ± 41.2	Time	0.40
Week 12	65.2 ± 35.3	56.4 ± 38.6	Group	0.31
			Time × group	0.92
Hyperaemic mean flow (ml/min)				
Week 0	491.9 ± 171.2	454.3 ± 179.1	Time	0.95
Week 12	526.3 ± 36.8	395.1 ± 134.6	Group	0.0271
			Time × group	0.035
Hyperaemic/baseline mean flow ratio				
Week 0	10.5 ± 6.1	14.2 ± 11.7	Time	0.70
Week 12	12.7 ± 14.6	10.8 ± 9.8	Group	0.73
			Time × group	0.20
Baseline local shear stress (dyne/cm^2^)				
Week 0	3.9 ± 2.3	3.2 ± 2.1	Time	0.28
Week 12	4.1 ± 2.3	3.8 ± 2.0	Group	0.33
			Time × group	0.68
Hyperaemic local shear stress (dyne/cm^2^)				
Week 0	28.2 ± 8.0	29.1 ± 8.3	Time	0.92
Week 12	27.6 ± 8.3	26.5 ± 7.8	Group	0.64
			Time × group	0.08

Mean values ± SD; 23–32 subjects in each treatment group, including all subjects with valid measurements at week 0 or 12. *P* denotes significant changes by repeated-measures MANOVA

**Table 4:** Assessment of endothelial function by treatment

	Week	Doxazosin	Ramipril	*P* by repeated-measures ANOVA	
FMD (%)	0	6.3 ± 4.4	5.3 ± 4.2	Time	0.34
	12	5.5 ± 3.1	4.5 ± 4.3	Group	0.75
	∆ 0 to 12	−0.3 ± 1.0	−1.1 ± 1.0	Time × Group	0.57
GTN (%)	0	15.5 ± 6.8	14.4 ± 7.0	Time	0.92
	12	14.4 ± 7.0	14.4 ± 6.9	Group	0.97
	∆ 0 to 12	−0.5 ± 1.3	0.3 ± 1.3	Time × Group	0.67
Endothelial functional index	0	0.47 ± 0.38	0.49 ± 0.56	Time	0.98
	12	0.51 ± 0.41	0.44 ± 0.64	Group	0.90
	∆ 0 to 12	0.07 ± 0.12	0.07 ± 0.12	Time × Group	0.42
Reflection index (%)	0	−7.3 ± 2.8	−6.8 ± 3.2	Time	0.68
	12	−6.6 ± 3.1	−7.7 ± 3.8	Group	0.54
	∆ 0 to 12	0.3 ± 0.9	−0.8 ± 1.0	Time × Group	0.43

Mean values ± SD for relative changes before and following drug treatment for 23–32 subjects in each treatment group, including all subjects with valid measurements at week 0 or 12, and absolute changes by treatment (∆, mean values ± SEM). *P* denotes significant changes by repeated-measures MANOVA. Adjustment for age; gender and smoking did not affect the results

Endothelial functional index was calculated as FMD/GTN as an index of endothelium dependent vasodilatation. Reflection index indicates the difference in pulse wave reflection before and after a subcutaneous injection of the beta-2 adrenoceptor agonist terbutaline

*FMD* flow mediated vasodilatation, *GTN* glycerine trinitrate

**Table 5:** Treatment effects on skin microcirculation, as assessed by laser Doppler fluxmetry

	Week	Doxazosin	Ramipril	*P* by repeated-measures ANOVA	
Ach peak flux	0	35.5 [21.0–61.7]	32.8 [17.8–62.4]	Time	0.96
	12	40.8 [20.1–66.5]	27.7 [19.0–52.6]	Group	0.35
Ach peak flux change week 0 to 12		0.6 ± 8.9	−2.2 ± 5.0	Time × group	0.82
Ach ∆ peak flux	0	29.0 [16.9–49.0]	25.1 [11.0–45.1]	Time	0.04
	12	35.0 [15.7–57.0]	23.1 [14.3–45.1]	Group	0.24
Ach ∆ peak flux change week 0 to 12		0.6 ± 8.1	−0.9 ± 4.4	Time × group	0.87
SNP peak flux	0	57.5 [33.4–77.9]	46.2 [33.9–83.8]	Time	0.38
	12	60.0 [44.0–81.7]	42.5 [26.4–87.4]	Group	0.23
SNP peak flux change week 0 to 12		−0.3 ± 10.3	−9.1 ± 8.4	Time × group	0.49
SNP ∆ peak flux	0	52.2 [27.4–71.2]	38.6 [27.6–75.2]	Time	0.27
	12	53.1 [34.7–72.9]	36.3 [17.7–83.1]	Group	0.24
SNP ∆ peak flux change week 0 to 12		−0.5 ± 9.7	−7.8 ± 7.8	Time × group	0.56
Ach/SNP peak flux	0	0.57 [0.40–0.96]	0.53 [0.41–0.98]	Time	0.28
	12	0.79 [0.29–1.41]	0.79 [0.38–1.28]	Group	0.46
Ach/SNP peak flux change week 0 to 12		0.26 ± 0.34	0.15 ± 0.20	Time × group	0.77
Ach/SNP ∆ peak flux	0	0.48 [0.38–0.99]	0.42 [0.30–0.91]	Time	0.16
	12	0.77 [0.23–1.51]	0.60 [0.29–1.49]	Group	0.24
Ach/SNP ∆ peak flux change week 0 to 12		0.57 ± 0.53	0.23 ± 0.23	Time × group	0.54
Max hyperaemia	0	61.9 [38.4–78.1]	58.0 [40.0–71.7]	Time	0.28
	12	54.5 [40.4–73.6]	56.8 [34.6–68.3]	Group	0.73
Max hyperaemia change week 0 to 12		−8.3 ± 7.9	−2.6 ± 4.5	Time × group	0.84
Max ∆ hyperaemia	0	53.5 [33.8–72.6]	47.6 [34.7–65.8]	Time	0.19
	12	49.0 [32.8–62.9]	52.9 [25.2–62.3]	Group	0.67
Max ∆ hyperaemia change week 0 to 12		−8.9 ± 7.4	−1.2 ± 4.2	Time × group	0.98

Median values and interquartiles or mean values ± SEM (for differences) from 27 to 33 subjects in each treatment group, including all subjects with valid measurements at week 0 or 12. Skin microvascular flux is expressed in arbitrary units. *P* denotes significant changes by repeated-measures ANOVA

∆ denotes the difference between rest and maximum values. Maximal hyperaemia was measured by local heating to 44 °C

*Ach* acetylcholine, *SNP* sodium nitroprusside

## Discussion

This study in patients with uncomplicated mild-to-moderate hypertension compared the effects of reducing noradrenergic sympathetic vascular tone by the alpha 1-adrenoceptor blocker doxazosin to blocking the RAAS by the ACE inhibitor ramipril to assess the possible influence of the RAAS on vascular structure and function beyond the effects on blood pressure. As expected, treatment with both doxazosin and ramipril for 12 weeks reduced brachial systolic and diastolic BP. This confirms an important role for both sympathetic vasoconstrictor nerve activity mediated by noradrenaline and for the RAAS through actions of angiotensin II in the control of vascular smooth muscle tone and BP in man. Furthermore, we found greater treatment induced reductions in aortic than in brachial systolic BP, and this did not differ between the two drugs. Our observations of larger reductions in central than in peripheral BP on ramipril are in agreement with findings with other ACE inhibitors [35, 36]. More important, our findings with doxazosin appear novel, as the effects of alpha-adrenoceptor blockers on central BP have not been well studied. Of note, beta-adrenoceptor blockers appear to have less effect on central BP, as compared to other drug classes [36]. Thus, both neurogenic sympathetic vasoconstriction and the RAAS are important for the control of central and peripheral BP.

Carotid-femoral PWV provides a good reflection of aortic stiffness, and antihypertensive treatment reduces PWV. Compared to doxazosin, ramipril reduced carotid-femoral PWV. These results persisted when accounted for potential confounding influence (i.e., mean arterial pressure, heart rate, height, age, and gender). This is in agreement with the previous observations that inhibition of the RAAS with ACE inhibitors or angiotensin receptor blockers [37–39] reduces (i.e., improves) aortic stiffness. However, the effects of doxazosin on indices of aortic stiffness in this study were minor. These results are novel, as the effects of alpha 1-adrenoceptor blockers on aortic stiffness have been little studied. One uncontrolled study in 11–15 Asian hypertensive patients suggested a low dose of doxazosin for 12 months to improve proximal aortic stiffness [40], and results reported in preliminary form suggested a reduction in PWV by doxazosin similar to that of a thiazide diuretic [41]. These results are in contrast to ours but may, at least in part, be due to differences in study design, population, and methodology. Thus, antihypertensive drug therapy by ramipril reduces aortic stiffness within 12 weeks of treatment, suggesting that the effects of ACE inhibitor therapy go beyond the effects of BP reduction, as also proposed by others [22].

Pulse pressure amplification, as compared to brachial pulse pressure, reflects pulsatile flow and gives information about central haemodynamic and microvascular damage. Thus, central pulse pressure is an indirect indicator of central aortic stiffness [42]. Accordingly, our findings with ramipril on carotid-femoral PWV were accompanied by consistent changes in other measures of pulsatile load, including reduced central pulse pressure, Aix (which mainly reflects peripheral resistance), and carotid-radial PWV (which reflects stiffness of peripheral conduit arteries), also when potential confounding influence (i.e. mean arterial pressure, heart rate, height, age, and gender) was considered. Thus, whereas alpha 1-adrenoceptor blockade seems to have little effect on arterial stiffness, ACE inhibitors may have additional beneficial effects beyond those related to BP reduction. This may be mediated by blocking the vasoconstrictor effects of angiotensin II, effects on structural vascular remodelling with hypertrophy and fibrosis, or by improved endothelial function [43, 44]. However, given the small effects by treatment on endothelial function (see below), alterations in endothelial function were less likely important for our results.

In this study, doxazosin or ramipril did not change post-ischemic FMD (considered to reflect mainly conduit artery endothelium dependent vasodilatation) or the forearm blood flow response to glyceryl trinitrate. These results were consistent also when the ratio FMD to glyceryl trinitrate (i.e., endothelial function index) was calculated, suggesting that drug treatment did not alter endothelial function. Consistent with these findings, endothelial dependent vasodilatation assessed by pulse wave analysis before and after beta 2-agonist stimulation (considered to reflect mainly resistance artery endothelium dependent vasodilatation) revealed little effect on reflection index by treatment with doxazosin or ramipril. In hypertensive patients with established coronary artery disease, suggesting more advanced atherosclerotic vascular disease, the ACE inhibitor quinapril, but not enalapril or the angiotensin receptor blocker losartan, improved endothelial function [45], whereas ramipril seemed to improve FMD in hypertensive patients in a dose-dependent manner [46]. Similarly, the effects on endothelial function by angiotensin receptor blockers in hypertension are not uniform [18, 19]. There is little reported on the effects of alpha 1-adrenoceptor blockers on endothelial function. A low dose of doxazosin for 12 months improved endothelial function in one small, uncontrolled study [40], whereas another small placebo controlled cross-over study demonstrated improved endothelial function by doxazosin [47].

We also investigated skin microcirculation by laser Doppler fluxmetry during iontophoresis of acetylcholine and sodium nitroprusside, and by local heating of the skin to evaluate treatment induced effects on endothelial function. This technique in the skin circulation has been shown sensitive to detect the early disturbances in endothelial function in man [33]. However, our results do not suggest alterations in skin microcirculation endothelial function by treatment with doxazosin or ramipril. Alpha 2-adrenoceptors are present on vascular smooth muscle endothelial cells and on the endothelium, where they mediate opposing effects on vascular tone. There is evidence that alpha 2-adrenoceptor agonist stimulation interfere with cutaneous microcirculation vasodilatation capacity after post-occlusive reactive hyperaemia, with increased production of nitric oxide [48]. However, little is published on the effects of alpha 1-adrenoceptor blockers and skin microcirculation in man [48, 49]. Concerning the RAAS, cross-sectional data suggest that skin microcirculation endothelial function was better in hypertensive patients treated with a combination of an ACE inhibitor and a diuretic (perindopril and indapamide), as compared to patients receiving treatment excluding an ACE inhibitor and/or a diuretic [50]. In contrast, endothelial function was improved similarly after 6 months treatment by the beta-adrenoceptor blocker metoprolol and the angiotensin receptor blocker olmesartan in another study [51].

Taken together, our results with three different validated non-invasive techniques to investigate endothelial function in various peripheral vascular beds were consistent. They suggest that antihypertensive treatment by reducing noradrenergic sympathetic vascular tone or by blocking the RAAS for 12 weeks does not influence endothelial dependent or independent vasodilation. However, these results should be interpreted with caution as the study population was relatively small and the variability of the methods for assessing endothelial function is not trivial. Nevertheless, this may be taken to suggest that our study population with uncomplicated mild-to-moderate hypertension had relatively normal endothelial function, where treatment induced improvement is difficult to detect. This is in consort with the observation that vascular remodelling develops at an earlier stage than endothelial dysfunction in hypertension [3]. More advanced hypertensive disease conditions with vascular dysfunction evident by coronary artery disease, diabetes, or other concomitant disease may be required to demonstrate improvement of endothelial dysfunction by antihypertensive treatment [45, 52].

There are several strengths to this double-blind randomized controlled study conducted in mostly never treated hypertensive patients. First, we compared the effects of blocking the RAAS with alpha 1-adrenoceptor blockade as an active control to assess the potential confounding effects of BP reduction by the ACE inhibitor. In addition, potential residual confounding effects of the magnitude of BP reduction were accounted for in the statistical analyses. However, the greater reduction in BP by ramipril than by doxazosin could have influenced our results on indices of vascular function. Second, we simultaneously studied several vascular beds. Third, we used several methods to evaluate endothelial function, and we included tests for both endothelium dependent and independent vasodilatation. However, there are important limitations to consider. First, the study population was of limited size, although this was justified by proper calculations of the required sample size. This may explain why we did not find changes in endothelial function by treatment. Second, the treatment period was 12 weeks and we cannot exclude that prolonged treatment could reveal other results. However, effects on BP and indices of aortic stiffness were already evident, and others have shown that changes in endothelial function can be demonstrated within 8–12 weeks in different study groups [46, 53]. Finally, although female sex hormones can influence endothelial function, we did not control for follicular/luteal phase of the menstrual cycle. However, very few patients were premenopausal and the potential confounding effects of menstrual cycle phase on our results are considered small.

In conclusion, doxazosin and ramipril similarly reduced central BP more than brachial BP, suggesting that both neurogenic sympathetic vasoconstriction and the RAAS are important for the control of central and peripheral BP. Ramipril reduced indices of aortic stiffness, suggesting that the effects of ACE inhibitor therapy go beyond the effects of BP reduction. However, we were unable to demonstrate an effect of treatment on endothelial function. Evidence of endothelial dysfunction and possible improvement by antihypertensive treatment might require more advanced hypertensive disease.
